# Experiences accessing nutritious foods and perceptions of nutritional support needs among pregnant and post‐partum mothers with low income in the United States

**DOI:** 10.1111/mcn.13660

**Published:** 2024-05-29

**Authors:** Jessie Benson, Matthew DeVries, Skye McLaurin‐Jiang, Christine D. Garner

**Affiliations:** ^1^ School of Medicine Texas Tech University Health Sciences Center Amarillo Texas USA; ^2^ Phoenix Children's Pediatric Residency Program Alliance Phoenix Arizona USA; ^3^ Department of Pediatrics Texas Tech University Health Sciences Center Amarillo Texas USA; ^4^ InfantRisk Center Texas Tech University Health Sciences Center Amarillo Texas USA; ^5^ Department of Obstetrics and Gynecology Texas Tech University Health Sciences Center Amarillo Texas United States

**Keywords:** food assistance, maternal nutrition, meal programme, post‐partum period, pregnancy, qualitative, social determinants

## Abstract

Access to nutritious foods, a social determinant of health, contributes to disparities in maternal and infant health outcomes such as mental health, breastfeeding intensity and cardiometabolic risk. This study explored perceived nutrition access and intake among pregnant or post‐partum women eligible for Medicaid. Qualitative, semistructured interviews were conducted with 18 women who were either currently pregnant (*n* = 4) or up to 12 months post‐partum (*n* = 14) in 2021–2022. Mothers spoke English (*n* = 11) or Spanish (*n* = 7) and lived in the Texas Panhandle. Interviews were audio‐recorded, transcribed, translated (Spanish to English) and verified. Two or more researchers coded each interview until consensus was reached using thematic analysis with ATLAS.ti software. The study revealed five drivers for nutrition access. (1) Social factors influenced nutrition; those with less support expressed limited ability to eat healthfully. (2) The Women, Infants and Children program was perceived as a helpful resource for some, while others faced challenges obtaining it. (3) Stress was bidirectionally related to unhealthy food choices, with food sometimes used as a coping mechanism. (4) Mothers prioritized their babies and others and had limited ability and time to prepare healthy meals. (5) Most participants felt they received inadequate nutrition guidance from their healthcare providers. Participants provided positive responses to a proposed nutritious home‐delivered meal intervention. Low‐income women may experience nutritional challenges specific to this life stage. Interventions that reduce stress and burden of household tasks (e.g. cooking) and improve education and access to nutritious foods may improve mothers' ability to consume nutritious foods.

## Key messages

- Pregnant and post‐partum women desire to eat nutritious foods for their own health and that of their infants.
- More support is needed for women in the post‐partum period as prioritization of others and the stressful transition of caring for a newborn hinder women from maintaining healthy eating habits.
- Pregnant and post‐partum women want focused nutritional guidance from their healthcare providers.
- A home‐delivered, nutritious meal programme may be an acceptable intervention to help alleviate the nutritional barriers that are faced by pregnant and post‐partum women.

## Graphical abstract

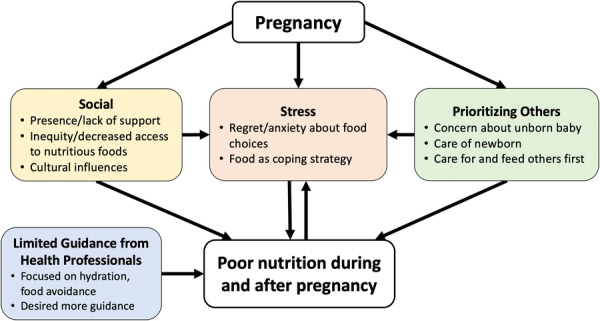

## 1 INTRODUCTION

In 2020, 28.6% of low‐income households in the United States were food insecure, having limited or uncertain access to adequate and nutritious foods, compared to the national average of 10.5% (U.S. Department of Agriculture, Economic Research Service, 2021). Food insecurity during pregnancy results in poorer periconceptional dietary quality and increased risk of complications such as gestational diabetes mellitus and weight gain (Laraia et al., 2010; Venkatesh et al., 2023). These conditions are associated with labour complications and adverse outcomes, including macrosomia, congenital heart defects and a higher risk of childhood obesity (Langley‐Evans et al., 2022). Post‐partum food insecurity is associated with maternal concerns of inadequate dietary intake and limited breastfeeding (Gross et al., 2019). Maternal food insecurity also increases the risk of post‐partum mental health disorders (Tarasuk et al., 2020).

The importance of nutrition during pregnancy and post‐partum has been well‐studied and received substantial attention. Adherence to a nutritious diet in the post‐partum period reduces the risk of post‐partum depression symptoms (Opie et al., 2020). Improved nutrition knowledge lowers post‐partum weight retention (Nuss et al., 2007). Furthermore, maternal diet may affect the nutritional composition of breast milk. While many human milk components are stable (e.g. lactose and protein) regardless of maternal diet, other components vary based on maternal diet, including docosahexaenoic acid and water‐soluble vitamins (Dror & Allen, 2018). Unfortunately, post‐partum women face many transitional challenges in caring for newborns that may prevent them from prioritizing healthful eating (Murray‐Davis et al., 2019). Jardí et al. (2019) found that consumption of nutritious foods decreased from early pregnancy to post‐partum despite increasing dietary and energy requirements for breastfeeding. This was hypothesized to be due to the desire to lose weight gained during pregnancy. Additional barriers to eating well during pregnancy and post‐partum include the financial cost of healthy food, the time associated with preparing meals, pregnancy symptoms like nausea and the need to prioritize other responsibilities (Grenier et al., 2021; Kovell et al., 2022; Murray‐Davis et al., 2019).

The Special Supplemental Nutrition Program for Women, Infants and Children (WIC) is a nutritional assistance programme aimed at preventing health consequences by providing nutrition education, healthy foods, referral services and breastfeeding support to low‐income populations at nutritional risk. Use of WIC benefits has been associated with improved maternal dietary quality, reduced infant mortality, reduced future risk of delivering low birthweight infants, developing maternal obesity and increased breastfeeding exclusivity and duration (Caan et al., 1987; Caulfield et al., 2022; Gross et al., 2023; Weinfield et al., 2020). Despite these benefits, only 51% of Texans eligible for WIC utilize its services due to many factors, including misconceptions about eligibility, language barriers and difficulty utilizing benefits.

Previous research has suggested that perinatal women may have unique needs for a supplemental intervention beyond WIC, such as healthy meal deliveries (Kovell et al., 2022). Medically tailored meal (MTM) programmes are a form of nutritious meal delivery intervention that has been studied among individuals with chronic medical conditions such as cirrhosis, diabetes and congestive heart failure (Belak et al., 2022; Kelly et al., 2023; Tapper et al., 2020). They have been shown to improve dietary quality, increase mental health‐related quality of life and reduce stress and burden of disease management (Berkowitz et al., 2019, 2020). More recently, MTM studies have been conducted among peripartum women with gestational diabetes (Huang et al., 2024). The objective of this qualitative study was to understand food preferences, dietary behaviours and nutritional support needs among peripartum women with low income to tailor nutrition interventions for this population.

## 2 METHODS

A qualitative study of pregnant and post‐partum women was conducted in a mid‐sized city (population of 201,234) situated in a rural area of the Texas Panhandle, where 34% of the population identified as Hispanic/Latino ethnicity (United States Census Bureau, 2022). Nutrition resources for perinatal mothers with low income in this region included WIC, one food bank and grocery stores located in several neighbourhoods, although absent in certain underserved neighbourhoods.

Qualitative methods used for this study followed those described by Braun and Clarke (2006), which are frequently used in applied research settings. This study was approved by the Institutional Review Board at Texas Tech University Health Sciences Center in Amarillo, TX. Reporting for this study follows the Standards for Reporting Qualitative Research (O'Brien et al., 2014).

### 2.1 Sampling

The focus population for this study was mothers who were pregnant or less than 12 months post‐partum and identified as eligible for public insurance (Medicaid) or WIC. Participants were recruited using flyers in obstetrics and gynecology clinics, the WIC office, local social media posts and chain referrals. Flyers and social media posts directed individuals to a brief Qualtrics survey with additional information about the study and collected demographic information to determine eligibility. To participate, individuals had to be pregnant or less than 12 months post‐partum and speak either English or Spanish; 44 English speakers and nine Spanish speakers responded who met the inclusion criteria. Eligible participants were contacted by the study team and scheduled for a focus group or individual interview.

The initial project design had a target sample size of 24–30 and intended to utilize focus groups of 6–10 people. However, due to limited resources, time constraints and substantial difficulties in scheduling focus groups with our population, we adjusted our data collection strategy to a combination of interviews and mini‐focus groups to maximize the number of participants. Sampling was purposive to obtain both English‐ and Spanish‐speaking individuals.

### 2.2 Data collection procedures

Through a comprehensive review of the literature, consultation with clinical social workers and input from clinical experts, we developed an interview guide to elicit mothers' perceptions about perinatal nutrition and recommendations for how a supplemental nutrition intervention could most effectively address the needs of new mothers (Table 1). Two licensed clinical social workers (LCSWs) who were experienced with the target population and trained in group facilitation conducted focus groups and interviews in English or Spanish. One of the LCSWs had prior experience conducting focus groups for research, and the other received training from the research team before data collection; the research team and LCSWs met regularly to review processes and address issues that arose during data collection. The facilitators had no relationships or interactions with the study participants before the interview.

**Table 1:** Qualitative interview domains, questions and probes related to nutrition during and after pregnancy.

Domains	Questions	Probes
Nutrition during pregnancy	What guidance did you receive about what to eat while you were pregnant? Did you feel like you were able to eat the right kinds of foods? What types of foods did you think that you ate more of or too much of?	What types of foods did you think you needed more of? What were some challenges with getting or eating these foods? What were some of the reasons for eating these foods? What would have helped you?
WIC	Did or do you participate in WIC? How did you learn about WIC? What was it like signing up for and participating in WIC?	Tell me about your experiences using WIC benefits. What were your experiences with obtaining food through WIC? What were your experiences with preparing or consuming WIC foods?
Nutrition after pregnancy	Let's have a conversation about eating and nutrition after pregnancy. In the first couple of months after your baby was born… What was it like obtaining food and meals to eat? Were these ‘right kinds of foods’ different or the same as during pregnancy? What were some of the challenges you faced with getting food/meals? What guidance did you receive about nutrition after your baby was born?	Did you feel like you were able to get the ‘right kinds of foods’ after your baby was born? What were some of the challenges you faced with obtaining these foods after your baby was born? What are some of the things that would have helped you?
Social support	How did you feel about the support you received in the first months after your baby was born?	Where did most of your support come from? Did you feel like you had the support you wanted?
Proposed nutritious meal delivery intervention	This programme would provide healthy meals in the third trimester of pregnancy and up to 12 weeks after delivering their babies. These meals would be delivered to mothers' homes two times per week. What do you think of this idea? What would make this more appealing? What concerns would you have about this programme?	How do you think your family would feel about your involvement in such a programme? Friends? What did you think about these meals? (process of heating, accessibility for busy moms, taste, portion sizes?) How would you feel if the menu was a preset menu with rotating items versus options that you could choose from? What would you prefer for the structure of meals for this programme‐7 single meals + seven snacks or six single meals + one meal for two?

Abbreviation: WIC, Women, Infants and Children.

One goal of this study was to obtain information to guide the design of a home‐delivered nutritious meal programme called MamaMeals. Before participation in a focus group or interview, each participant received and consumed two such meals, so that they could provide feedback about them. Data were gathered through mini‐focus groups (up to four participants) or individual interviews (for three participants) between October 2021 and April 2022. Concepts that arose in focus group sessions did not differ from one‐on‐one interviews. Due to evolving restrictions related to the COVID‐19 pandemic regarding in‐person events, interviews were conducted via Zoom in the first months of the study. In‐person interviews began in March 2022.

Questions centred around food access, nutritional barriers during pregnancy and post‐partum, social needs, experiences with the sample meals provided and thoughts regarding a prepared meal intervention (Table 1). At the time of the session, participants reported demographic information. Sessions ranged from 30 to 100 min and averaged 62 min. Participants received a $50 gift card.

### 2.3 Data analysis

English interviews were held over Zoom, recorded and auto‐transcribed by Zoom. Spanish interviews were held in person, recorded, transcribed and translated into English by a certified bilingual clinician not involved in the analysis. All interviews were audio‐recorded, deidentified and double‐checked by a second investigator for accuracy. Raw narrative data were entered into ATLAS.ti 9 software to organize and perform thematic analysis of each transcript (Braun & Clarke, 2006).

Before coding, two team members read each transcript in its entirety. An initial list of broad concepts was created, upon which codes were developed and themes were identified. C. D. G. and S. M.‐J. created the initial coding scheme from the first three interviews. Subsequently, each transcript was coded independently by at least two of four researchers (J. B., M. D., C. D. G. and S. M.‐J.). Following each iteration of coding for each transcript, team members revisited the codes, adding or removing codes as deemed necessary by the team until a consensus was reached regarding each quotation and selected code. Major themes and subthemes were discussed and agreed upon by the team.

### 2.4 Trustworthiness

The research team and interviewers convened following each interview session to reflect on important concepts that emerged. Following the initial sessions, concerns about questions and wording of the interview guide were addressed to improve data collection (McMahon & Winch, 2018). Credibility was enhanced through peer debriefing, whereby emerging themes and findings were discussed with professionals not directly involved in this research (Lincoln & Guba, 1985). Researcher bias was minimized through reflections and frequent discussion among team members where assumptions could be challenged (neutrality). Evidence that contradicted our interpretations was sought out, and negative cases were identified through coding and analysis to further enhance trustworthiness (Lincoln & Guba, 1985).

## 3 RESULTS

### 3.1 Participants

Eighteen mothers participated in this study (Table 2). Participants spoke either English (*n* = 11) or Spanish (*n* = 7). Nearly two‐thirds of participants reported Hispanic ethnicity (*n* = 11); the remainder reported as White and non‐Hispanic (*n* = 7). The average age of participants was 31 years old (range 22–40). Fourteen were within 12 months post‐partum, and four participants were pregnant. Six out of the 18 total participants were primiparous.

**Table 2:** Characteristics of peripartum mothers participating in focus groups or individual interviews (*N* = 18).

Characteristic	*n* (%)^a^
Age, years^a^	
Mean (range)	31 (23–40)
Race/ethnicity	
White, Non‐Hispanic	7 (39)
Hispanic	11 (61)
Language	
English	11 (61)
Spanish	7 (39)
Pregnant/post‐partum	
Pregnant	4 (22)
Post‐partum	14 (78)
Parity	
Primiparous	6 (33)
Multiparous	12 (67)
Individually interviewed	3 (17)

a Data presented as *n* (%) except where designated.

### 3.2 Overview of themes

**Figure 1:**
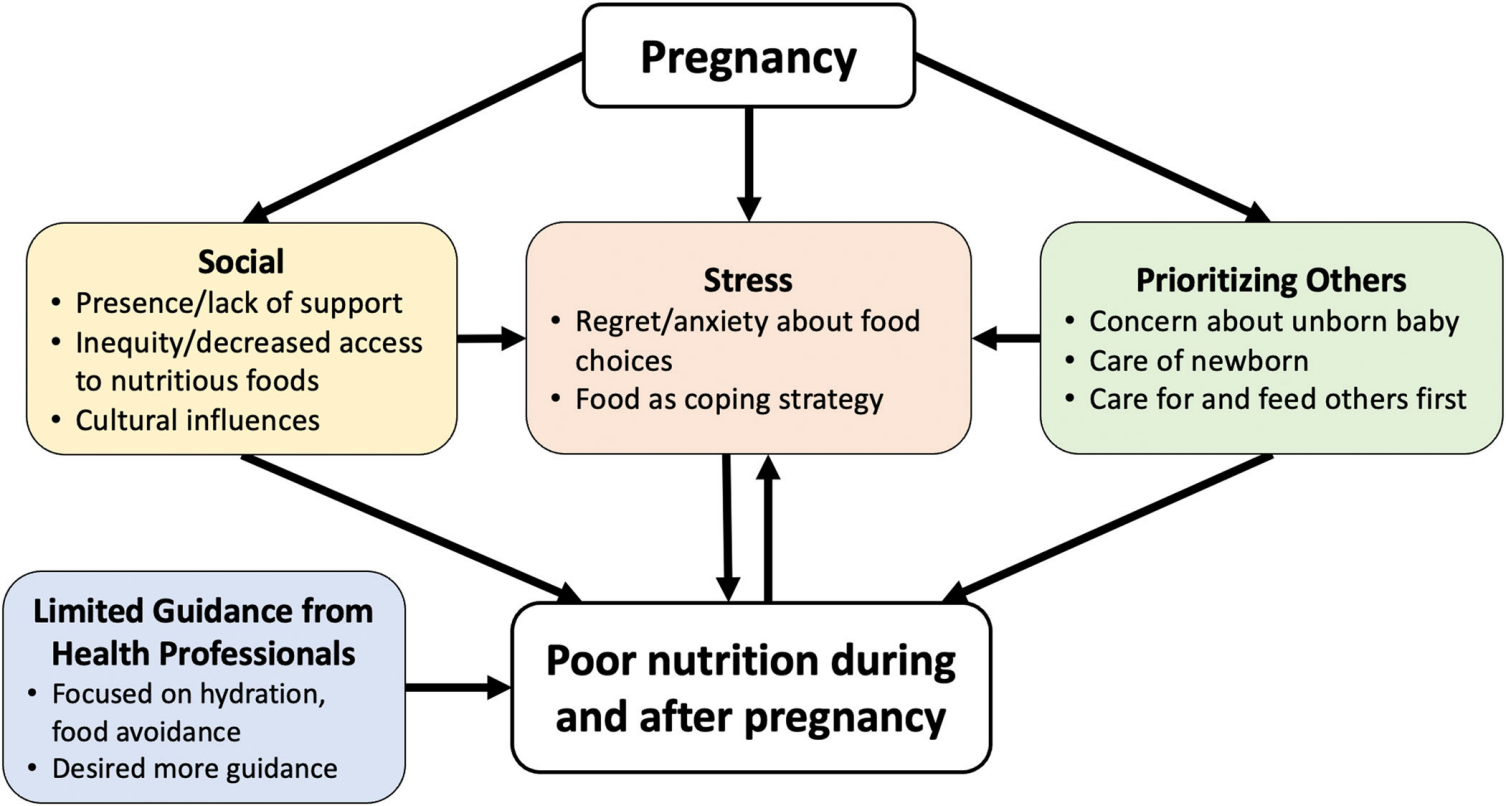
Factors contributing to poor nutrition during and after pregnancy. Social support, stress, prioritization of others and lack of professional guidance influenced access to and consumption of nutritious foods during and after pregnancy.

Five themes, each with subthemes, emerged related to mothers' experiences accessing nutritious foods and their perceptions about nutrition during and after pregnancy (Figure 1). Participants also provided overwhelmingly positive responses about a proposed home‐delivered nutritious meal programme and provided perspectives on what would be most beneficial or preferred. Quotations below are denoted by the focus group number (e.g. FG01), the participant number (e.g. P01), and the language in which that session was conducted (English or Spanish).

Theme 1 *Social support influences nutrition.*

*The presence or lack of social support affected participants' abilities to access or consume nutritious foods. Some participants received tangible support such as meal preparation by family and friends, childcare and help around the house. This support was perceived as helpful and decreased stress about household responsibilities. Those with nearby family or a supportive partner described obtaining meals more easily because ‘it was a team effort’ (FG02, P03, English). Women with strong, close‐knit support described that their needs were being met, which improved opportunities for good nutrition.*

‘His parents [brought] us food… because if not, we were just forgetting [to cook], because it was like oh, we need to focus on the little human’. (FG01, P01, English)

Some mothers described a lack of social support which made it difficult to obtain healthy foods. Participants whose spouses had unpredictable work schedules described challenges with preparing meals because there was no one to help with the children. Participants with no significant other described more challenges obtaining and consuming nutritious foods due to a lack of tangible assistance and financial strain.

Mothers who had strong support systems did not receive support as much or as long as they had hoped. One mother described good support immediately after her baby was born, but this help ceased quickly.

‘I have… a lot of family nearby… We had people bring us meals. And that was fantastic, you know, but that was like maybe the first week or so… It's not like everything just snaps back to normal’. (FG02, P03, English)

Familial and cultural influences impacted maternal food choices. Some Hispanic mothers shared how particular cultural beliefs about foods influenced their thoughts regarding nutrition and diet while pregnant, including ‘eating for two’ (FG08, P14, Spanish), that specific foods may affect how the baby looks, and a perceived need to ‘drink a lot of milk to make milk’ (FG06, P08, English). Some mothers of Hispanic descent described how their family or friends would comment about their weight gain or body changes, resulting in added stress about body image.

‘[During pregnancy] they tell me, you are very fat, you are gaining a lot of weight… they give a lot of importance to your weight and your image, and that is not what one should be worried about’. (FG08, P13, Spanish)

Theme 2 *WIC is a support for some, while others struggle to obtain it.*

*While all study participants qualified for WIC, a range of perceptions existed about its usefulness. Many WIC participants felt that it was a helpful resource as it relieved the financial burdens of obtaining nutritious foods and made it easier to obtain fruits, vegetables and dairy products.*

‘If it wasn't for WIC, and also I was working at the time, I don't think I would have been able to keep myself fed’. (FG01, P01, English)

However, WIC was not always perceived as convenient as it did not alleviate the challenges of having to go to the grocery store, which was difficult with a newborn or several kids in tow, and was feared to expose their newborns to germs. Mothers also described experiencing technical difficulties with WIC, such as inconsistencies in the foods identified as WIC‐eligible and issues with card readers processing their cards at checkout.

In the Spanish‐speaking focus groups, the challenges of obtaining WIC were perceived to outweigh its benefits. One mother voiced that it was ‘easier to not have WIC, than have WIC’ (FG08, P14, Spanish). The process of enroling in WIC was described as time‐consuming, and the paperwork could be onerous:

‘They ask for the income and my husband does not make the same every check and they pay him cash, and it is a lot of paperwork’. (FG08, P12, Spanish)

For women with other children, obtaining childcare to attend appointments and educational sessions posed an additional burden.

Theme 3 *Stress was bidirectionally related to unhealthy food choices.*

*Stress related to a mothers' own nutritional status and food choices was common. Mothers believed and understood that the pregnancy and post‐partum periods were integral for both their own health and their children's, and this could create pressure or cause guilt. Women felt pressure from themselves, their families, spouses and medical professionals to be extra vigilant about their food choices due to concerns about how food choices would affect their unborn child or their newborn if breastfeeding.*

‘I would get anxious that I could not eat [healthy food], I always try to eat healthy… but [while pregnant] with him I struggled a lot, a lot’. (FG09, P16, Spanish)

Other women felt pressure to eat healthy because of how eating might result in regret or perceived excessive weight gain. Despite motivation to make healthier food choices, many moms found it difficult due to a lack of energy, time and demands from other areas of their lives. The fatigue of pregnancy led many mothers to seek out prepackaged meals or dine out. Women described anxiety and ‘regret’ around their food choices, leading to excess stress.

‘I would always crave [fast food]. I was like I don't need to be eating this fried food, but then I would go on walks … I felt like me walking I could reward myself with that. But yeah no. That was a bad idea’. (FG04, P06, English)

The combination of pregnancy‐related stressors and other social factors post‐partum, including caring for other children, relationships with partners and family and balancing work or errands, led some to use food as a coping mechanism. Some women expressed that eating sweets or junk food gave them a sense of comfort or relieved stress, even when they recognized they were eating unhealthy foods.

‘[M]y choices weren't the healthiest because of how I was feeling emotionally. So I feel like food was kind of like my comfort’. (FG04, P06, English)

In some cases, using food as a coping mechanism was not a conscious choice but based on an emotional need that was not recognized until later. Other women embraced using food as a coping strategy. These women recognized that the post‐partum period was challenging and that excessive worry about what they ate would cause undue stress that they would rather avoid.

‘I'm like, you know what, I will eat whatever it takes to get me through this day and keep me in a good mood’. (FG06, P09, English)

Theme 4 *Prioritization of baby and other family members.*

*Mothers were conscientious about providing for their children and family members but often did not have the time or energy to prioritize themselves. Even when mothers ate nutritiously, it was often because they felt accountable for helping their developing fetus grow rather than for the sake of taking care of themselves. They believed that the food they consumed would affect their baby and wanted to make food choices accordingly. Interestingly, some women did not prioritize their nutrition during pregnancy but were conscious about making healthier choices post‐partum because they were breastfeeding.*

‘I was eating much more fried food, while I was pregnant. Um but like once you had your baby, you were thinking about like, how is it impacting’. (FG04, P06, English)

Post‐partum, responsibilities for the infant and family members hindered mothers' abilities to eat well. It was also challenging to accomplish tasks necessary for meal preparation, like grocery shopping or cooking, because the infant would cry or other children would need her attention. As the primary caregivers of the household, some mothers were often so focused on caring for their families and infants that their own well‐being fell by the wayside.

‘I had to make food for my kids, my husband and me, and then with a baby that wants to always be with you. It is very frustrating at the beginning because you have all these feelings together sometimes during the pregnancy, and after pregnancy you also want to cry’. (FG08, P12, Spanish)

Newborn care often took priority, causing mothers to be too tired or have little motivation to prepare food for themselves. Several described forgetting to eat because of this.

Mothers felt that their social support people also tended to prioritize the newborn. For one mom, this was particularly salient:

‘… a lot of people don't ask like how's mom doing … you just want to hold the baby… everything that [the mom is] going through now, like that's a lot. And it is about mom; like it has to be, because if mom's not okay baby's not going to be okay…’ (FG05, P07, English)

Theme 5 *Nutrition guidance from health care professionals was inadequate.*

*During pregnancy, participants expected to receive information about nutrition, especially from health care professionals (HCPs), but felt that this guidance was limited and desired more detailed nutrition information. Advice received from HCPs primarily focused on foods to avoid and increasing hydration. Participants described receiving more advice from midwives than physicians. Insufficient guidance led pregnant women to seek out additional nutrition information from online resources, family members and/or friends.*

‘I didn't receive a whole lot of information from my doctor… it wasn't like a whole lot of useful information… So what I've kind of been doing is, uh, my own research mostly, like Pinterest and Google’. (FG05, P07, English)

Several women who were dissatisfied with advice from HCPs felt that the WIC programme provided valuable guidance.

‘[T]hat's been I guess the most advice that I've gotten, is from [WIC]’. (FG05, P07, English)

Participants gave insight into the guidance they would have liked to receive. Some participants expressed interest in seeing a nutritionist who might provide frequent check‐ins specific to nutrition at their health visits. There was also interest in receiving education on cooking nutritious meals, portion control and eating healthier. One mom wished to have received specific, targeted information on what foods were best during certain stages of development. Multiparous participants described receiving some guidance during their first pregnancy but little or no guidance after. They desired to receive nutrition information during each pregnancy, as a refresher or for an update on nutrition guidelines:

‘I wish they would give everybody a guide every time, even if they've had kids before’. (FG01, P02, English)

### 3.3 Feedback on the proposed MamaMeals intervention

Overall, participants expressed overwhelmingly positive feedback about a proposed home‐delivered meal programme as well as the meals that they received and consumed.

Participants liked the home‐delivery aspect of the programme as it provided convenience for overwhelmed mothers and was a source of nutritious food even when tired or preoccupied with other responsibilities. They appreciated that receiving the food was not an extra chore. It also circumvented the numerous barriers mothers face when leaving their homes with an infant or taking multiple kids grocery shopping.

‘…it feels like you're going to have that extra support…getting food into your home, especially when you can't go to the store because you're just too tired or you just don't have the time or the baby's sick or something’. (FG07, P10, English)

Nearly all participants preferred having a choice in the meals that they received. Reasons for this centred around the importance of autonomy but also included pickiness or being ‘not very adventurous when it comes to food’ and accommodating food allergies. One participant described that having autonomy in this way was important because of how limited she felt with food options during pregnancy.

‘…[H]aving that autonomy to be able to choose what it is that you want to eat and put in your body that week is important, too…especially when [moms] feel like they've already been so limited because you can't eat so much while pregnant’. (FG05, P07, English)

Mothers expressed that having home‐delivered nutritious meals would reduce stress by decreasing the cooking burden, freeing up time for other tasks and feeding their children. Having a readily accessible, easy‐to‐heat up, preprepared meal provided a healthy, nutritious meal without adding time and energy to prepare one. However, this did not alleviate the concern of having to prepare meals for other family members. Although the meals were intended for the mothers, some mothers used the meals as a quick and easy way to feed their kids, giving moms time to themselves or to focus on other tasks.

‘I will end up sharing with my kids or I'll just completely give it my kids because, honestly, like I don't feel like cooking anything and I'm like, here, I'll snack on something a little bit later if it just makes them be quiet, or I have a moment of peace or I could get something done’. (FG07, P11, English)

## 4 DISCUSSION

The pregnant and post‐partum women with low income in our study desired to eat nutritiously, but often felt unable to do so. The increased stress of caring for their newborn, in addition to household responsibilities, left mothers prioritizing other family members' nutrition over their own. Women relied on WIC and HCPs for support, but some experienced challenges obtaining WIC benefits and limited nutritional guidance from HCPs. Our participants believed that a programme that delivers fresh, nutritious, prepared meals would improve their ability to consume healthy foods during and after pregnancy.

Social support was a key factor in maternal healthy eating in our study. Social support has been identified as a crucial factor influencing maternal role development (Emmanuel et al., 2008). Lubker Cornish and Roberts Dobie (2018) identified that in the post‐partum period, social support in the forms of emotional, informational and instrumental support was particularly helpful; the latter two were endorsed by our study participants as beneficial in helping them eat nutritiously. Unfortunately, the amount of instrumental support mothers receive in the post‐partum period may fall short compared to needs, consistent with the experiences of our study participants who described that more attention was often given to the baby rather than the mother (Lubker Cornish and Roberts Dobie, 2018). Even with good social support, healthy eating was especially challenging for mothers during the early post‐partum period. During pregnancy, mothers were motivated to eat nutritiously as they wanted to make food choices that positively impacted their baby's health, a finding also observed by McNamara et al. (2022). However, while new mothers desired to eat nutritiously post‐partum, the new responsibilities of caring for a newborn on top of other day‐to‐day demands were stressful. Mothers prioritized other responsibilities over taking care of themselves, leading to incongruence between the mother's expectations for healthy eating and reality (Murray‐Davis et al., 2019). Lack of energy and time often led mothers to prioritize food convenience over food quality, which has been observed by others (McNamara et al., 2022).

Many participants conveyed that the WIC programme facilitated healthy eating by providing needed access to nutritious foods and nutrition education. Despite maternal and neonatal benefits, a significant proportion of WIC‐eligible women do not participate in WIC. This has been attributed in part to various administrative barriers of WIC, including paperwork, time, gathering documentation and overall inconvenience (Davis et al., 2022). These challenges were endorsed in our study, especially among Spanish‐speaking participants. Some perceived that WIC created additional burdens as paperwork and attending WIC meetings prevented them from accomplishing household duties. Consistent with findings from Davis et al. (2022), our participants also described circumstances that limited the usefulness of WIC, including a lack of knowledge of WIC‐eligible foods and difficulty in physical access to WIC services. Subsequent studies should explore differences in WIC accessibility by user language.

In our study, cultural beliefs were a barrier to obtaining adequate nutrition during pregnancy and post‐partum. Others have reported similar findings. In Pakistan, the importance of consuming ‘hot’ versus ‘cold’ foods prevented women from adequate intake of protein‐rich foods during pregnancy (Asim et al., 2022). Women also limited their food consumption to avoid eating in ‘excess’ for fear of having a larger baby and needing a Caesarean section (Asim et al., 2022). Gill et al. (2004) reported that Mexican Americans described specific foods to avoid or eat while breastfeeding to prevent the milk from going bad or to increase milk production. Among our Hispanic participants, cultural beliefs tied the consumption of specific foods to direct impacts on the baby; for example, eating tacos will result in a ‘baby born with a taco face’, and the ‘baby will have a cold if you drink cold things’. While participants did not necessarily agree with these cultural beliefs and statements from their families, they still influenced how mothers viewed nutrition and what they ate. This is consistent with research from Mexico on pregnant and breastfeeding women of low‐middle socioeconomic classes, which showed that even individuals with adequate knowledge about a cultural myth still act on the myth (Sámano et al., 2020).

Participants in our study expected to receive nutritional counselling from their HCPs but were dissatisfied with the limited information they received. Similarly, other qualitative studies have reported women feeling ‘underwhelmed’ with guidance received, leading them to make nutrition choices based on instinct or Internet searches (Daigle Millan et al., 2022). Many HCPs report low confidence in providing nutritional and exercise guidance to pregnant or post‐partum women due to a lack of knowledge and training (Lee et al., 2018; Murray‐Davis et al., 2019); this may explain why the guidance received by our participants was limited to hydration and food avoidance.

Interestingly, the few participants who received care from a midwife tended to describe the guidance they received as more satisfactory. Some evidence suggests that midwives may provide more nutritional guidance after delivery than physicians (Farrar et al., 2013). Similarly, Meulenbroeks et al. (2021) reported that 60% of midwives discussed maternal diet at the first prenatal appointment compared to 24% of obstetricians. However, a study of Australian midwives showed that while most midwives provided nutritional counselling to pregnant women, only half had received proper training (Arrish et al., 2016). When counselling on maternal weight gain, both physicians and midwives face barriers of subject sensitivity, lack of knowledge of proper resources and financial limitations, with physicians having an extra barrier of time constraint in their clinic visits (Murray‐Davis et al., 2020). Prenatally, American College of Obstetricians and Gynecologists (ACOG) has guidelines for addressing gestational weight gain and obesity but has no specific nutritional guidance for the post‐partum period (American College of Obstetricians and Gynecologists, 2018). Having individualized nutritional guidance is important, particularly in older pregnant women, and may reduce pregnancy complications and improve both maternal and child outcomes (Li et al., 2021).

Participants in our study believed that a novel home‐delivered nutritious meal programme would be very beneficial during and after pregnancy. They expected such a programme would reduce stress due to decreased cooking burden, increased time for other chores and feeding other children. Previous research on MTMs suggested that accommodating taste preferences and cultural acceptability is attractive to participants (Berkowitz et al., 2020). Our findings echoed the importance of having the choice in selecting foods; this may be especially important perinatally as some women may experience food aversions. Furthermore, having a choice in meals was perceived to provide pregnant women with a sense of autonomy during a time when they felt limited by their food options due to pregnancy.

Convenience, portion size and easy preparation of meals were also important to our participants and have been attractive to participants in other meal programmes (Kelly et al., 2023). Our findings further suggest that meal intervention programmes may expand palates by introducing new nutritious foods and stimulating ideas for meal preparation after the programme has ended. Combining meal programmes with nutrition education and other lifestyle interventions may further alleviate nutritional barriers and health risks women face during pregnancy or the post‐partum period. One‐third of women with normal prepregnancy body mass index become overweight or obese at 1 year post‐partum (Endres et al., 2015). Post‐partum weight retention contributes to obesity and overall long‐term cardiovascular risk, further highlighting a role for nutritious food interventions perinatally.

## 5 LIMITATIONS

There were several limitations to our study. First, while our sample included WIC‐eligible mothers, it may not have included the most marginalized, such as those experiencing homelessness or severe food insecurity. Future studies should aim to better understand the perspectives of the most underserved perinatal individuals. Second, all English‐language interviews were completed before those in Spanish. The impact of cultural beliefs did not emerge as an important concept until the Spanish interviews; thus, we were unable to fully explore the influence of culture on nutrition among those who spoke English. The collection of more extensive demographic and behavioural information would have provided more context for the interpretation of our findings. Future studies would benefit from a better understanding of prior food intake, behaviours such as cooking, medical conditions, pregnancy complications and perceived stress and social support.

The use of focus groups may have limited participants' willingness to share their perspectives; alternatively, focus groups stimulated discussion and allowed participants to build on each other's opinions. While it was necessary to provide meals to participants to elicit their opinions about them, there is a potential concern that providing free meals before participation may have had an impact on their responses. Due to COVID‐19 precautions, some interviews were conducted virtually and, therefore, subject to technical difficulties, including lagging speech, overlapping conversation and background noises.

## 6 CONCLUSION

Pregnant and post‐partum women with low‐income desire to eat nutritious foods both for their own health and that of their infants. Unfortunately, they experience many barriers to eating nutritiously, including stress, low energy, competing demands for their time and a lack of tangible and informational support. Interventions that reduce stress and the burden of household tasks and provide adequate education may improve mothers' ability to consume nutritious foods. A home‐delivered, nutritious meal intervention may alleviate some burden of responsibilities and support the mom directly in consuming nutritious foods while serving as a guide to healthful eating and easing transitions from pregnancy to post‐partum.

## AUTHOR CONTRIBUTIONS

Christine D. Garner and Skye McLaurin‐Jiang designed the research study. Jessie Benson, Matthew DeVries, Christine D. Garner and Skye McLaurin‐Jiang analysed the data. Jessie Benson and Matthew DeVries created the initial draft of the manuscript. Christine D. Garner and Skye McLaurin‐Jiang provided critical revisions for important intellectual content. All authors approved the final version of the manuscript.

## CONFLICT OF INTEREST STATEMENT

The authors declare no conflict of interest.

## Data Availability

Data that support the findings of this study are available on request from the corresponding author. The data are not publicly available due to privacy or ethical restrictions.
